# Bromide toxicosis (bromism) secondary to a decreased chloride intake after dietary transition in a dog with idiopathic epilepsy: a case report

**DOI:** 10.1186/s12917-021-02959-x

**Published:** 2021-07-22

**Authors:** Marco Fantinati, Nathalie Priymenko, Maud Debreuque

**Affiliations:** 1 Department of Nutrition, University of Toulouse, ENVT, Toulouse, France; 2 Department of Internal Medicine/ Neurology, University of Toulouse, ENVT, Toulouse, France

**Keywords:** Bromism, Bromide toxicosis, Potassium bromide, Chloride, Dog, Epilepsy, Nutrition

## Abstract

**Background:**

Bromide is a halide ion of the element bromine usually administered in the form of potassium salt as monotherapy or add-on treatment in epileptic dogs. It is excreted unchanged in the urine and undergoes tubular reabsorption in competition with chloride. Thus, dietary chloride content affects serum bromide concentrations. This is the first published clinical report of bromide toxicosis secondary to a dietary modification of chloride content in an epileptic dog treated with potassium bromide.

**Case presentation:**

A 3-year-old 55-kg neutered male Tibetan Mastiff was evaluated because of a 1-month history of progressive signs including ataxia, lethargy and behaviour changes. The dog was successfully treated for idiopathic epilepsy since the age of 1-year-old with phenobarbital and potassium bromide. Two months prior to presentation, the owners decided to change the dog’s diet without veterinary advice. Physical examination was unremarkable. A 12-kg weight gain was recorded since last follow-up (8 months). Neurological examination revealed severe symmetric 4-limbs ataxia with altered vigilance and intermittent episodes of hyperactivity and aggressive behaviour without significant abnormality of cranial nerves. Serum bromide concentration was high and increased by 103 % since last follow-up. Nutritional evaluation revealed a 53 % decrease of chloride content in the diet before and after dietary transition. Bromide toxicosis was suspected, due to bromide reduced clearance secondary to the decreased dietary chloride content. Potassium bromide treatment was lowered by 15 % without further dietary changes. Neurologic signs progressively improved over the next month, without any seizure. After two months, the serum bromide concentration lowered to the same level measured before dietary modification. After four months, neurological examination was unremarkable.

**Conclusions:**

Dietary chloride content can directly influence serum bromide concentrations, therefore affecting seizure control or contributing to unexpected adverse effects. In the present case, a reduction in chloride intake markedly increased serum bromide concentrations causing bromism. Dietary changes should be avoided in dogs treated with potassium bromide to maintain stable serum bromide levels.

## Background

Bromide toxicosis, also known as “bromism”, is a rare intoxication resulting from the ingestion of excessive bromide-containing compounds [1]. Reports of poisoning have been described both in dogs and humans treated with bromide salts (e.g. potassium or sodium bromide) [2–4]. Humans usually exhibit non-specific symptoms, such as anorexia, weight loss and nausea, but may develop life-threatening neuropsychiatric (e.g. ataxia, tremor, delirium or acute psychosis), dermatologic and gastrointestinal signs [4]. Comparatively, dogs may show similar adverse effects to high bromide salts intake, but main clinical manifestations of true bromism are of neurologic nature, including alterations of consciousness, ataxia, and upper and lower motor neuron tetra- and paraparesis [1, 5, 6].

Despite only recently receiving the Food and Drug Administration (FDA) conditional approval,^1^ the halide salt potassium bromide (KBr) has long been used as monotherapy or add-on antiepileptic drug in the treatment of seizures associated with idiopathic epilepsy (IE) in dogs [7–9]. Although its precise mechanism of action is still unknown, bromide ions have a preferential movement through gamma-aminobutyric acid-activated chloride channels, resulting in hyperpolarization of neurons, therefore raising the threshold to seizure initiation [10]. Furthermore, bromide does not undergo hepatic metabolism, is completely filtered unchanged by the glomerulus and competes with chloride for tubular reabsorption [11]. As a consequence, the rate of renal elimination of bromide varies directly with chloride intake: high dietary chloride will increase bromide excretion, therefore shortening its half-life; on the contrary, low dietary chloride will decrease bromide excretion and prolong its half-life [9].

Feeding dogs different dry diets formulated with increasing chloride contents has showed to significantly affect serum bromide concentrations: the higher was the chloride content in the diet, the lower was the bromide concentration in the serum [12]. Chloride content in dry diets may have huge differences from one product to another depending on formulation. Pet foods designed for dissolving struvite or prevent calcium oxalate stones may contain the highest concentration of chloride on the market. Reason behind this is the supplementation of sodium chloride during formulation aimed to achieve sodium concentrations capable of promoting urine dilution and decrease supersaturation [13]. These pet foods may be defined as “high-chloride diets”, and clinical proof has already been published describing how their introduction in dogs already receiving KBr treatment, has the potential to increase bromide clearance to a point where its serum therapeutic range cannot be maintained [14].

This clinical report describes a 3-year-old Tibetan Mastiff diagnosed with IE, and treated with a combination of phenobarbital (PB) and KBr, developing signs of bromide toxicosis after an unprescribed dietary transition to a dry pet food with a lower chloride content compared to the previously eaten diet. There is no previously published proof showing a direct relationship between the modification of dietary chloride content and the clinical appearance of bromism. Furthermore, both the dry diets eaten by the dog where not formulated neither as high- or low-chloride diets, highlighting how regardless of the type of diet, it is the delta between the two that clinically affects bromide clearance.

## Case presentation

A 3-year-old 55-kg neutered male Tibetan Mastiff was evaluated because of a 1-month history of progressive signs including ataxia, lethargy and behaviour changes. At 6-months-old, the dog manifested clusters of generalized seizures. A diagnosis of IE was suspected because of normal blood analyses, unremarkable brain computed tomography (CT) scan and cerebrospinal fluid (CSF) analysis. At first, PB^2^ was prescribed as monotherapy (5 mg/kg, PO, q 12 h). Because of the appearance of adverse effects early after treatment initiation, the pet-owners arbitrarily decided to lower the PB dose (2.3 mg/kg/ PO, q 12 h). Four months later, because of cluster seizures still occurring every 7 to 20 days, KBr^3^ was introduced as additional antiepileptic drug (40 mg/kg, PO, q 24 h). After seven months of combined therapy, seizure clusters resolved with a single episode of seizure reported. Despite the improvement of seizure control, the dog presented mild hyporexia and marked 4-limbs ataxia as a consequence of the combined anticonvulsant therapy. Because the drugs’ secondary effects were deemed unacceptable by the pet-owners, KBr dosage was decreased by 17 % (33 mg/kg, PO, q 24 h). Four months later (eight months before presentation), a seizure-free status was achieved, paralleled by a reduction of the aforementioned adverse effects. Ataxia was still present, therefore KBr treatment was again lowered by 15 % (28 mg/kg, PO, q 24 h). Complete resolution of neurological signs was progressively achieved within a month. Six months prior to presentation, according to the owners’ perception, the dog’s gait, behaviour and appetite were considered completely normal. A 6-kg weight gain was reported.

Because of a history of chronic diarrhoea, at the time of the first reduction of KBr, the dog was transitioned to a dry hydrolysed diet (Pet Food A). Gastrointestinal signs were positively affected by the highly digestible kibbles. Prompted by almost one year with no diarrhoea, two months before presentation, the owners decided to switch the dog’s back to an over-the-counter adult dry diet (Pet food B).

**Fig. 1:**
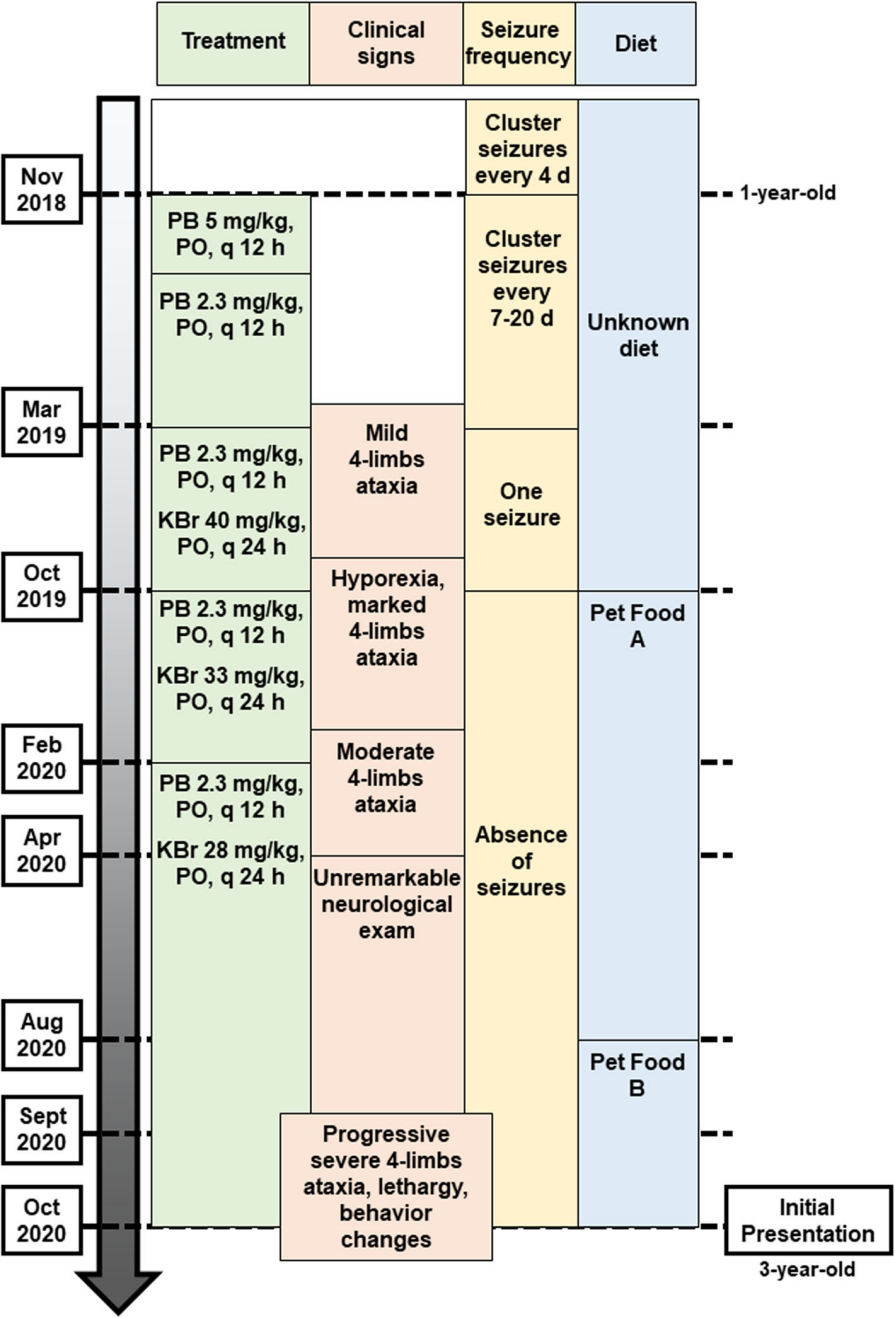
Graphical representation of the present case’s timeline before presentation

Antiepileptic treatment, clinical signs, seizure frequency, and diet prior to presentation, are summarized in Fig. 1.

Physical examination at presentation was unremarkable. Over an 8-month period, a total 12-kg weight gain was registered. Body condition was scored 6 on the 9-point scale [15]. Neurological examination revealed an abnormal gait, with severe symmetric 4-limbs ataxia and frequent falls. The dog showed behavioural changes during consultation, such as aggression (e.g. attempt to bite when handled) and disorientation. Furthermore, outbursts of hyperactivity were displayed through attempts to jump and climb on doors and windows.

Postural reactions were abnormal on all four limbs, with pelvic limbs more affected (in particular hopping reactions). Spinal reflexes were normal. Cranial nerves examination did not reveal any significant abnormality. Neurological examination was consistent with upper motor neuron (UMN) tetraparesis.

The pet-owners did not consent to a brain magnetic resonance imaging (MRI), which was proposed in completion to the diagnostic work-up that previously brought to the presumptive diagnosis of IE.

Present antiepileptic treatment consisted in the combination of PB (2.3 mg/kg, PO, q 12 h) and KBr (28 mg/kg, PO, q 24 h).

Results of complete blood count and serum biochemical analysis were within reference ranges, except for a high serum alkaline phosphatase activity (1450 U/L; reference range, 20 to 155 U/L), which is a common finding with long-term administration of PB. Markers of renal function were normal and similar to previous measurements (Creatinine, 65.9 [59.2–71.7] µmol/L; reference range, 44 to 133 µmol/L). Serum chloride concentration was markedly high (> 128 mmol/L; reference range, 115 to 128 mmol/L) paralleled by a normal serum sodium level (143 mmol/L; reference range, 138 to 148 mmol/L). Pseudohyperchloremia was suspected secondary to KBr treatment.

Serum PB concentration was 20 µg/mL (therapeutic range, 15 to 35 µg/mL) [9, 16], while serum bromide was 2800 mg/L. The latter showed a 103 % increase since last follow-up 8 months ago (1380 mg/L). Analyses of serum PB and bromide were always processed at the same laboratory^4^.

Analysis of chloride content in both Pet Food A and B was obtained via potentiometric titration at an external certified private laboratory^5^. Results showed 1.91 and 1.01 g/1000 kcal ME (0.46 and 0.24 g/MJ ME) of chloride in Pet Food A and B, respectively (Table 1). Considering that daily energy intake did not significantly differ from before and after dietary transition, a 53 % decrease of dietary chloride was found between the two dry diets.

**Table 1:** Metabolisable energy and main analytical constituents of the study diets. Nutrient values are displayed on an as fed and DM basis (%) and according to the diet’s energy density (g/1000 kcal)

	Pet food A		Pet food B	
**ME (kcal/kg)**^a^	4079 (17.07 MJ/kg)		3835 (16.05 MJ/kg)	
	**% as fed (% DM basis)**	**g/1000 kcal (g/MJ)**	**% as fed (% DM basis)**	**g/1000 kcal (g/MJ)**
**Crude protein**	21 (23.2)	51.48 (12.30)	30 (32.8)	78.23 (18.70)
**Crude fat**	19 (21.0)	46.58 (11.13)	18 (19.7)	46.94 (11.22)
**Crude fibre**	1.1 (1.2)	2.70 (0.65)	3.5 (3.8)	9.13 (2.18)
**Crude ash**	6.1 (6.7)	14.95 (3.57)	8.5 (9.3)	22.16 (5.30)
**NFE**	43.3 (47.9)	106.15 (25.37)	31.5 (34.4)	82.14 (19.63)
**Omega-3**	0.95 (1.05)	2.33 (0.56)	2.8 (3.06)	7.30 (1.74)
**Omega-6**	4.46 (4.93)	10.93 (2.61)	1.6 (1.75)	4.17 (0.97)
**Calcium**	0.8 (0.88)	1.96 (0.47)	1.6 (1.75)	4.17 (0.97)
**Phosphorus**	0.6 (0.66)	1.47 (0.35)	1.2 (1.31)	3.13 (0.75)
**Sodium**	0.46^b^ (0.51)	1.13 (0.27)	0.30^b^ (0.33)	0.78 (0.19)
**Chloride**	0.78^c^ (0.86)	1.91 (0.46)	0.39^c^ (0.42)	1.01 (0.24)

^a^Predicted by the National Research Council (2006) equation based upon crude fibre.

^b^Determined by flame atomic absorption spectrometry (laboratory Eurofins Analytics France, Nantes, France).

^c^Determined by potentiometric titration with silver nitrate (laboratory Eurofins Analytics France, Nantes, France).

*ME* metabolisable energy; *NFE* nitrogen-free extract

Bromide toxicosis (bromism) was suspected, due to bromide reduced clearance secondary to the decreased dietary chloride content.

Hospitalization and parenteral infusion with saline solution were proposed as first line treatment to increase bromide renal clearance. The owners refused the approach, knowing that the dog will have suffered being alone in the stressful environment of the hospital.

To address the clinical manifestations, it was decided to lower by a 15 % KBr (24 mg/kg, PO, q 24 h) treatment while leaving unchanged PB. The 1-year period without seizure was considered enough to justify the avoidance of a novel antiepileptic drug. Pet-owners were advised to strictly avoid any further dietary change.

**Fig. 2:**
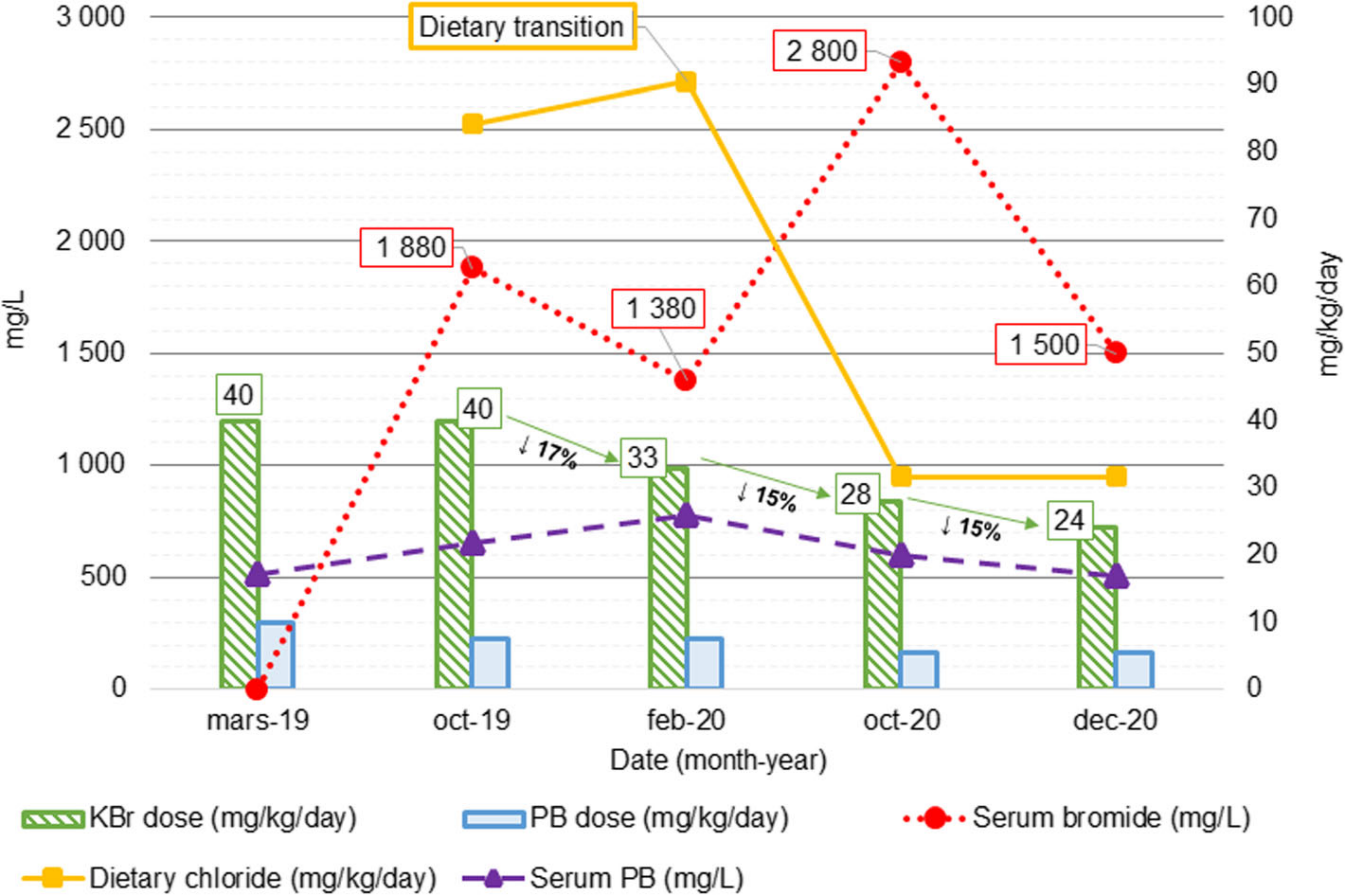
Relationship between the antiepileptic drugs’ dose, PB and bromide serum concentrations and the dietary chloride intake

Ataxia and behaviour changes progressively improved without seizure recurrence. After two months, mild symmetric ataxia was still observed, but otherwise neurological examination was unremarkable. Serum bromide concentration lowered (1500 mg/L) to the same level measured before dietary modification, while serum PB kept stable (17 µg/ml) (Fig. 2).

Despite lowered since first evaluation, serum alkaline phosphatase remained moderately high (650 U/L; reference range, 20 to 155 U/L).

According to the pet-owners, four months after reduction of the KBr treatment, the dog’s gait and behaviour were completely normal and no seizure was recorded.

## Discussion and conclusions

To the authors’ knowledge, this is the first published clinical report describing bromism secondary to dietary transition in an apileptic dog treated with KBr.

Hinting to a diagnosis of bromism were both the clinical observation of ataxia and the neurological examination consistent with UMN tetraparesis. In a review of 1298 medical records of dogs diagnosed with IE and treated with bromide salts, Rossmeisl and Inzana found that the prevalence of bromism in such patients was 2 % (n = 31). As in the present case, main signs of poisoning were UMN tetraparesis (26 %) and an ataxic gait (52 %) [1].

Other finding supporting the diagnosis was the markedly increased serum chloride concentration (> 128 mmol/L) paralleled by a normal level of sodium (143 mmol/L). In humans, this pattern is considered a hallmark of bromide intoxication [4]. Despite the actual circulating chloride level is likely to be closer to normal, pseudohyperchloremia can occur depending on which analytical method is used by the laboratory. Some methods (e.g. ion-specific electrode or colorimetric) are unable to distinguish chloride from bromide; thus, the total halide ion concentration falsely elevates chloride measurements [17]. Contrary to findings in epileptic children, the degree of pseudohyperchloremia cannot be used in dogs as indirect estimator of bromide concentration [18].

Final confirmation of bromism was brought by the marked increase (103 %) of serum bromide concentration (2800 mg/L) during monitoring of KBr treatment at presentation. According to Trepanier et al., when KBr is combined to PB for seizure control in dogs, the reasonable therapeutic range for serum bromide concentration is 810 to 2400 mg/L [7]. As in the present clinical report, all published cases of bromism in dogs showed serum bromide concentrations above the proposed therapeutic range: Rossmeisl and Inzana reported a mean value of 3700 mg/L, while Yohn et al. and Nichols et al., 2700 and 3120 mg/L, respectively [1–3].

Dosing errors, severe dehydration, and exposure to halothane anaesthesia are cited in literature as possible causes of high serum bromide concentration [19]. A more plausible cause, directly related to the pharmacokinetic of KBr, is renal insufficiency. In a clinical report by Nichols et al., an epileptic dog treated with a combination of PB (1.9 mg/kg, PO, q 12 h) and KBr (29 mg/kg, PO, q 24 h) at a dosage not far from the one used in the present study, developed signs of bromism (e.g. ataxia, posterior paresis) revealing a serum bromide concentration of 3120 mg/L. Monitoring of renal function found both creatinine and blood urea nitrogen (BUN) within reference ranges, but measurement of glomerular filtration rate via endogenous creatinine clearance uncovered renal insufficiency [3]. In the present case, serum creatinine and urea were in the reference ranges with normal urine specific gravity. Because the abrupt increase of serum bromide concentration was coincidental with a weight gain and an unprescribed dietary transition, renal insufficiency was not pursued as possible underlying cause, and glomerular filtration or other markers of kidney function (e.g. symmetric dimethylarginine) were not assessed.

As previously said, dietary chloride may also increase competition for tubular reabsorption between chloride and bromide. In a clinical trial by Trepanier et al., three groups of dogs (n = 12) were fed dry pet foods with increasing chloride content (0.2, 0.4 and 1.3 % DM basis). Daily dietary chloride intake was estimated for each group (32, 64 and 193 mg/kg, respectively) and after two weeks of acclimation to the novel diets, all dogs received KBr treatment (20 mg/kg, PO, q 24 h, providing 14 mg/kg of bromide) for an 8-week period. Results showed that the rate of bromide elimination was directly increased by the increasing dietary chloride content (mean half-life of 69, 46 and 24 days, respectively). Estimates of the optimal daily bromide doses required to keep serum levels above 1000 mg/L, showed statistically significant differences (P = 0.002) between the groups. Dogs fed 1.3 % chloride (43 ± 13 mg/kg) requiring almost two- (22 ± 3 mg/kg) or three-fold (15 ± 4 mg/kg) the dose estimated for those fed 0.4 and 0.2 % chloride, respectively [12].

Illustrating the impact of dietary chloride content on serum bromide, a clinical report by Shaw et al. described the case of an epileptic dog treated with PB (3 mg/kg, PO, q 12 h) and KBr (20 mg/kg, PO, q 24 h) manifesting a cluster of seizures after three months of seizure-free status because of dietary change. One week before symptoms, due to the development of cystic calculi, a dry pet food formulated to dissolve struvite uroliths was introduced as main diet. Due to the high-chloride content (1.18 % DM basis) of the latter, bromide renal excretion was enhanced and the serum therapeutic bromide concentration could not be maintained (from 1100 mg/L to 410 mg/L) [14].

The same mechanisms detailed in these studies about the effect of enteral chloride intake on bromide excretion, may explain the clinical manifestations of bromism in the present case. After almost one year of seizure-free status, the dog developed signs of bromide toxicosis two months from a dietary transition. Pet Food A (chloride 0.86 % DM basis) was not a high-chloride diet per se. Nonetheless, Pet Food B (chloride 0.42 % DM basis) contained less than half (53 %) its chloride content. Considering the 12-kg weight gain, possibly due to reduced physical activity and polyphagia secondary to the antiepileptic drugs, the average chloride intake was 84.46 and 31.64 mg/kg/day while eating Pet Food A and B, respectively.

The present case highlights how regardless the type of diet (i.e. high-, moderate- or low-chloride), in case of a dietary transition in dogs treated with KBr, it is the delta between the chloride content of the two diets that eventually will cause a modification in the bromide clearance.

Chloride content of pet foods can vary greatly, and most of this mineral comes from supplementation of chloride-containing salts (e.g. ammonium chloride, sodium chloride) or vitamins (e.g. choline chloride) during manufacturing [20].

Despite some manufactures may voluntarily declare chloride concentration in pet foods formulated for specific dietary purposes (e.g. uroliths management), the EU and US legislations do not require this nutrient declaration between the analytical constituents on the label. In our case, the lack of information about chloride content on the label was, in fact, the reason for which the feed analysis became necessary.

As well as enteral chloride, parenteral chloride has been showed to affect bromide renal clearance in dogs, representing the basic treatment for bromide toxicosis. In the previously cited report by Nichols et al., treatment with saline solution (110 mL/kg, IV, q 24 h; serum bromide from 3120 mg/L to 1980 mg/L after 24 h) was successful in detoxifying from bromide poisoning, contradicting previous results by Yohn et al., who failed to quickly reduce serum bromide with a lower dose of saline solution (60 mL/kg, IV, q 24 h; serum bromide from 2700 mg/L to 2600 mg/L after 24 h) [3]. In the attempt to define a protocol to rapidly reduce bromide concentrations, Fukunaga and colleagues assessed the parenteral infusion of three different solutions (saline, Na^+^ 154 mmol/L and Cl^−^ 154 mmol/L; Ringer lactate, Na^+^ 131 mmol/L and Cl^−^ 110 mmol/L; maintenance, Na^+^ 35 mmol/L and Cl^−^ 35 mmol/L) in dogs with steady-state serum bromide levels. The authors found that higher sodium and chloride contents in infusion fluids brought to greater change from baseline, and higher renal clearance. Serum bromide was reduced by 14.24 % and urine bromide by 17.63 % when saline or lactated Ringer’s solutions were infused (10 ml/kg/h, IV, for 5 h) [21]. No consensus has yet been reached on bromism treatment in epileptic dogs treated with bromide salts. Clinical reports published on the subject, describe a reduction or discontinuation of KBr treatment, frequently paralleled by IV administration of saline solution (0.9 % NaCl) and, sometime, furosemide [1, 2]. Although this therapeutic approach may fasten clinical improvement, breakthrough seizures may occur [1, 2]. Thus, treatment of bromism should be tailored for each individual animal based on seizure frequency and severity of the clinical manifestations.

The present clinical report showed that in dogs treated with KBr, it is truly fundamental the avoidance of any dietary modification without previous precise quantification of the daily chloride intake, a step necessary to adjust the KBr dose according to the new diet chloride content. Recommendation of avoidance of unprescribed dietary transition is mandatory for pet-owners of epileptic dogs treated with KBr.

## Data Availability

All data generated or analysed during this study are included in this published article.

## Abbreviations

DM: Dry matter
IE: Idiopathic epilepsy
KBr: Potassium bromide
ME: Metabolisable energy
PB: Phenobarbital
UMN: Upper motor neuron

## Footnotes

1 U.S. Food & Drug Administration (FDA) official website. Conditional approval of KBr for dogs – an innovative approach. Content current as of the 12th of February 2021: https://www.fda.gov/animal-veterinary/resources-you/conditional-approval-potassium-bromide-dogs-innovative-approach.

2 Phenoleptil® 100 mg, Dechra Pharmaceuticals, Skipton, UK.

3 Solibrom-BR® 600 mg, Laboratoire TVM, Lempdes, France.

4 University of Toulouse Veterinary Hospital internal laboratory, Toulouse, France.

5 Eurofins Analytics France, Nantes, France.
